# BC RNA Mislocalization in the Fragile X Premutation

**DOI:** 10.1523/ENEURO.0091-18.2018

**Published:** 2018-04-19

**Authors:** Ilham A. Muslimov, Taesun Eom, Anna Iacoangeli, Shih-Chieh Chuang, Renate K. Hukema, Rob Willemsen, Dimitre G. Stefanov, Robert K. S. Wong, Henri Tiedge

**Affiliations:** 1 The Robert F. Furchgott Center for Neural and Behavioral Science, State University of New York Downstate Medical Center, Brooklyn, New York 11203; 2 Department of Physiology and Pharmacology, State University of New York Downstate Medical Center, Brooklyn, New York 11203; 3 Statistical Design and Analysis, Research Division, State University of New York Downstate Medical Center, Brooklyn, New York 11203; 4 Department of Neurology, State University of New York Downstate Medical Center, Brooklyn, New York 11203; 5 Department of Clinical Genetics, Erasmus Medical Center, 3000 CA Rotterdam, The Netherlands

**Keywords:** CGG repeats, cognitive impairment, epileptiform activity, regulatory RNAs, RNA localization

## Abstract

Fragile X premutation disorder is caused by CGG triplet repeat expansions in the 5′ untranslated region of FMR1 mRNA. The question of how expanded CGG repeats cause disease is a subject of continuing debate. Our work indicates that CGG-repeat structures compete with regulatory BC1 RNA for access to RNA transport factor hnRNP A2. As a result, BC1 RNA is mislocalized in vivo, as its synapto-dendritic presence is severely diminished in brains of CGG-repeat knock-in animals (a premutation mouse model). Lack of BC1 RNA is known to cause seizure activity and cognitive dysfunction. Our working hypothesis thus predicted that absence, or significantly reduced presence, of BC1 RNA in synapto-dendritic domains of premutation animal neurons would engender cognate phenotypic alterations. Testing this prediction, we established epileptogenic susceptibility and cognitive impairments as major phenotypic abnormalities of CGG premutation mice. In CA3 hippocampal neurons of such animals, synaptic release of glutamate elicits neuronal hyperexcitability in the form of group I metabotropic glutamate receptor–dependent prolonged epileptiform discharges. CGG-repeat knock-in animals are susceptible to sound-induced seizures and are cognitively impaired as revealed in the Attentional Set Shift Task. These phenotypic disturbances occur in young-adult premutation animals, indicating that a neurodevelopmental deficit is an early-initial manifestation of the disorder. The data are consistent with the notion that RNA mislocalization can contribute to pathogenesis.

## Significance Statement

The fragile X premutation finds expression in two distinct disease manifestations. Young premutation carriers may present with seizure activity and cognitive disturbances, whereas advanced-age patients may experience intention tremor and gait ataxia, a condition known as FXTAS. In contrast to FXTAS, the early-onset phase of fragile X premutation disorder remains poorly understood. We discovered that in brains of CGG-repeat knock-in animals (a premutation mouse model), regulatory BC1 RNA is mislocalized, as its presence at synapto-dendritic domains is severely diminished. Lack of BC1 RNA is known to cause epileptogenesis and cognitive dysfunction, and we report that such phenotypic alterations are hallmarks of young premutation animals. The data are congruous with a potential role of RNA localization impairments in pathogenesis.

## Introduction

In mammalian neurons, translational control mechanisms play important roles in the input-dependent regulation of local protein synthesis in postsynaptic dendritic microdomains (Gkogkas et al., 2010; Darnell, 2011; Iacoangeli and Tiedge, 2013; Eom et al., 2018). A key requirement for such mechanisms is the targeted delivery of requisite components, including select species of RNA, to synapto-dendritic neuronal domains (Doyle and Kiebler, 2011; Iacoangeli and Tiedge, 2013; Eom et al., 2018).

Regulatory BC RNAs are small cytoplasmic RNAs (scRNAs) that operate in the translational regulation of local protein repertoires in neurons (Iacoangeli and Tiedge, 2013; Eom et al., 2014, 2018). Dendritic transport of BC RNAs is mediated by cis-acting dendritic targeting elements (DTEs) that reside in 5′ stem-loop domains (Muslimov et al., 2006, 2011). BC RNA DTEs are double-stranded structural RNA motifs that carry spatial codes (Doyle and Kiebler, 2011) in the form of noncanonical (i.e., non–Watson-Crick) nucleotide base pairings (Muslimov et al., 2006, 2011). These architectural motifs are specifically recognized by RNA transport factor hnRNP A2 in interactions that are required for BC RNA dendritic delivery (Muslimov et al., 2006, 2011; Iacoangeli and Tiedge, 2013).

Fragile X premutation disorder, a common inherited disability, is caused by CGG trinucleotide repeat expansions, in the range of 55–200 repeat units, in the 5′ untranslated region (UTR) of FMR1 mRNA (Oostra and Willemsen, 2009; Santoro et al., 2012; Nelson et al., 2013). The FMR1 premutation is estimated to occur with a mean frequency of 11.7 per 10,000 (1/885) in males and 34.4 per 10,000 (1/291) in females (Sherman and Hunter, 2017). The disorder finds expression in both early- and late-onset clinical manifestations (Nelson et al., 2013; Hagerman et al., 2016). Early-onset neurodevelopmental symptoms include seizure activity, cognitive impairment, and autism-spectrum disorder (ASD; Aziz et al., 2003; Farzin et al., 2006; Clifford et al., 2007; Bailey et al., 2008; Hagerman et al., 2010, 2016; Chonchaiya et al., 2012; Hagerman, 2013). A late-onset neurodegenerative phase of fragile X premutation disorder, the fragile X-associated tremor/ataxia syndrome (FXTAS), is characterized by gait ataxia, intention tremor, and cognitive decline (Oostra and Willemsen, 2009; Hagerman, 2013; Nelson et al., 2013). FXTAS may represent the final stage of a disease process that is initiated much earlier, i.e., during childhood (Hagerman et al., 2010, 2016; Hagerman, 2013).

How does CGG-repeat RNA cause cellular dysfunction and disease? FMR1 mRNA premutation CGG repeats can sequester RNA binding proteins (RBPs), as a consequence making them unavailable to perform their normal cellular functions (Swanson and Orr, 2007; Hagerman et al., 2010; Hagerman, 2013; Nelson et al., 2013). CGG repeat interactions with several RBPs, including hnRNP A2, Purα, and Sam68, have been causally implicated in fragile X premutation disorder (Jin et al., 2007; Sofola et al., 2007; Sellier et al., 2010; Nelson et al., 2013). Other mechanistic scenarios, not necessarily mutually exclusive, have been advanced for the FMR1 premutation, including models in which expanded CGG repeats are translated into a polyglycine-containing protein (Todd et al., 2013; Kearse et al., 2016; Sellier et al., 2017).

We hypothesized that premutation FMR1 mRNA is pathogenic because its 5′ CGG-repeat stem-loop structures feature noncanonical nucleotide interactions that are similar to those in 5′ BC RNA dendritic targeting elements (DTEs). Accordingly, our working hypothesis made two specific predictions: (1) CGG RNA competition for hnRNP A2, which is required for BC1 RNA transport (Muslimov et al., 2006, 2011), will compromise synapto-dendritic delivery of this regulatory RNA in vivo; and (2) because lack of BC1 RNA causes epileptogenic susceptibility and cognitive impairment (Zhong et al., 2009; Chung et al., 2017; Iacoangeli et al., 2017), our working hypothesis predicted that absence or significantly reduced presence of BC1 RNA in synapto-dendritic domains would trigger cognate phenotypic alterations in CGG premutation animals. We further predicted that such alterations would occur as early onset (i.e., preceding late-onset FXTAS), representing a significant but hitherto enigmatic initial manifestation of premutation pathogenesis (Hagerman et al., 2016).

## Materials and Methods

### EMSA competition analysis

We used plasmid pBCX607 to generate full-length rat BC1 RNA (Muslimov et al., 1997, 2011) and plasmid pUC57_BC200 to generate full-length human BC200 RNA. pUC57_BC200 was constructed by introducing the following insert (T7 promoter—BC200 RNA gene) into the EcoRV site of pUC57 (Genscript): 5′-TAATACGACTCACTATAGGCCGGGCGCGGTGGCTCACGCCTGTAATCCCAGCTCTCAGGGAGGCTAAGAGGCGGGAGGATAGCTTGAGCCCAGGAGTTCGAGACCTGCCTGGGCAATATAGCGAGACCCCGTTCTCCAGAAAAAGGAAAAAAAAAAACAAAAGACAAAAAAAAAATAAGCGTAACTTCCCTCAAAGCAACAACCCCCCCCCCCCTTTAAA-3′. Plasmid p(CGG)_105_ was used to generate (CGG)_105_ RNA (Muslimov et al., 2011).

Native PAGE gels were run on 8% polyacrylamide gels (ratio acrylamide/bisacrylamide 19:1) in 90 mm Tris-borate, pH 8.3, in the presence of 15 mm MgCl_2_ at room temperature for 12 h at 15 V (Muslimov et al., 2006, 2011). EMSA analysis was performed as described (Muslimov et al., 2006, 2011). ^32^P-labeled RNA in vitro transcripts (50,000 cpm per reaction) were heated for 10 min at 70°C, cooled for 5 min at room temperature, and incubated together with proteins in binding buffer (300 mm KCl, 5 mm MgCl_2_, 2 mm DTT, 5% glycerol, 20 mm Hepes, pH 7.6) for 20 min at room temperature.

For EMSA competition experiments, recombinant hnRNP A2 (100 nm) was pre-incubated with BC200 RNA or BC1 RNA at 37°C for 15 min, at which point (CGG)_105_ repeat RNA was added at a 1:3 molar ratio to the respective BC RNA. Aliquot samples were collected at the time points indicated in Fig. 2 and examined by native PAGE. Plasmid pET-9c (Munro et al., 1999) was used to express recombinant full-length hnRNP A2 (Muslimov et al., 2011).

Regulatory BC RNAs are relatively abundant in neurons. In vivo intracellular concentrations are estimated in the submicromolar range, as BC RNAs interact with eIFs 4A and 4B, initiation factors present in that concentration range (Chicurel and Harris, 1992; Hershey and Merrick, 2000; Lin et al., 2008). mRNAs are expressed at molar levels that are lower (typically by two orders of magnitude) than those of more abundant RBPs such as hnRNP A2 and those of regulatory RNAs such as BC RNAs. The question is thus raised how premutation CGG-repeat FMR1 mRNA can, in the cellular milieu of premutation neurons, effectively displace BC RNAs from hnRNP A2.

Two considerations are relevant in this context: (1) in murine and human premutation cells, levels of Fmr1/FMR1 mRNA are significantly increased, up to 8-fold in human carriers with CGG expansions in the 180-repeat range (Tassone et al., 2000; Brouwer et al., 2008); and (2) in CGG animals used here, the number of CGG repeat units is increased from a wild-type (WT) number of 8–10 to a premutation number of 180. We estimate that as a consequence, in vivo concentrations of CGG repeat units (the actual entities that successfully compete with BC RNAs for access to hnRNP A2) are elevated by about two orders of magnitude in (CGG)_180_ cells, relative to WT cells. Our EMSA competition approaches were designed to mimic this situation in vitro with component concentrations in the 10–100-nm range, as described previously (Muslimov et al., 2011).

### Animals

(CGG)_n_ knock-in (KI) animals were initially generated by exchanging the murine (CGG)_8_ repeat with a human (CGG)_98_ repeat (Bontekoe et al., 2001). Subsequently, a line of (CGG)_180_ animals was established, using animals with 180 repeat units that had developed by spontaneously occurring intergenerational expansions in the colony (Brouwer et al., 2008). CGG and WT mice were on a mixed C57BL/6 and FVB/N genetic background. Male animals were used throughout. Work with animals was approved by the Institutional Animal Use and Care Committee of SUNY Downstate Medical Center.

### CGG-repeat lengths and FMRP expression levels

CGG animals were genotyped for CGG-repeat lengths as follows (Hukema and Oostra, 2013). Genomic DNA was extracted from tails of CGG and WT animals using the DNeasy Blood & Tissue Kit (Qiagen) according to the manufacturer’s instructions. CGG repeat length was determined by PCR using the KAPA2G Robust Hotstart PCR Kit (KAPA Biosystems). Approximately 500–1000 ng DNA was added to 25 μl of PCR mixture containing 0.4 μm of each primer, 250 μm of dNTPs, 2.5 m betaine (Sigma-Aldrich), and 1.25 U KAPA2G Hotstart. The forward primer sequence was 5′-CGGGCAGTGAAGCAAACG-3′, and the reverse primer sequence was 5′-CCAGCTCCTCCATCTTCTCG-3′. PCR steps were 5-min denaturation at 95°C, followed by 35 cycles of denaturation for 15 s at 95°C, annealing for 15 s at 55°C, and elongation for 1.5 min at 72°C. Final extension was performed for 5 min at 72°C. DNA samples were resolved on 1.5% agarose gels.

**Figure 1.**
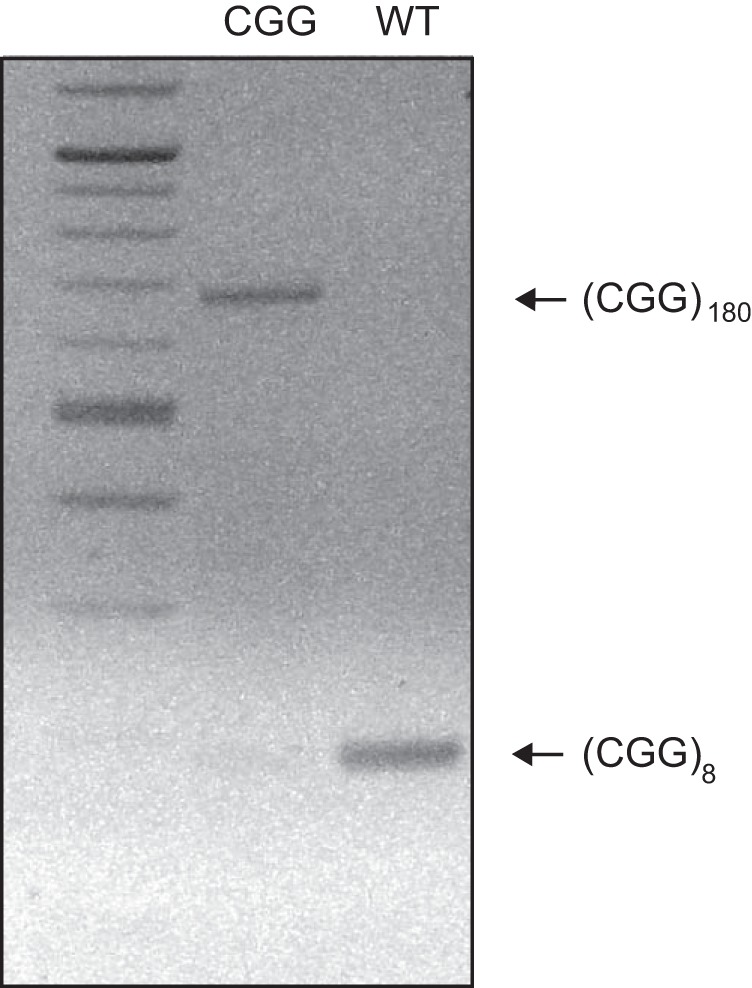
CGG repeat length genotyping. PCR was performed with genomic DNA isolated from animal tails, using primers specific for CGG repeats. Shown is an inverse image of an ethidium bromide–stained agarose gel of the PCR products. The results confirmed that a CGG mouse carried 180 CGG repeats (lane 2), whereas a WT mouse carried 8 CGG repeats (lane 3). A 100-bp DNA ladder was used in lane 1.

We observed CGG repeat length instability in our CGG animal colony, as reported earlier (Brouwer et al., 2008). Intergeneration and interanimal repeat length instability manifested in expansions as well as contractions. CGG-repeat lengths were established for both breeding colony animals and all experimental animals (in the latter cases *post hoc*, i.e., after completion of experiments). A validated (CGG)_180_ animal colony was maintained through CGG repeat length monitoring (Fig. 1) and coordinated breeding strategies. WT animals were confirmed as typically having 8 or 9 CGG repeats (Fig. 1). (CGG)_180_ animals were, for the purpose of this work, defined as animals with CGG-repeat lengths of 160–180 units. CGG animals with repeat lengths outside this range were excluded from experimental analysis.

Such exclusion was also mandated by FMRP expression level considerations. FMRP levels may be reduced, depending on CGG repeat length, in premutation human subjects and animal models (Feng et al., 1995; Brouwer et al., 2008; Iliff et al., 2013; Ludwig et al., 2014). A reduction in brain FMRP levels may contribute to premutation phenotypes and would therefore constitute a potential confound in the analysis (Ludwig et al., 2014; Renoux et al., 2014).

Work with CGG animals of various repeat lengths revealed a monotonic decrease of brain FMRP levels as a function of CGG repeat lengths ranging from 9 (WT) to 250 units (Ludwig et al., 2014). In agreement with these results, we found that average brain FMRP levels in (CGG)_180_ animals were ∼25% lower than respective average levels in age-, sex-, and strain-matched WT animals. Also in agreement with the earlier data (Ludwig et al., 2014), we observed substantial interanimal variability of brain FMRP levels among WT animals and especially among (CGG)_180_ animals. Brain FMRP levels of most (CGG)_180_ animals were within the range of WT animal levels. (CGG)_180_ animals whose brain FMRP levels were outside the WT range were not admitted into analysis. Brain FMRP levels were established *post hoc* for experimental animals. FMRP levels were determined by Western blot (see below) using antibody 4317 (Cell Signaling Technology, # 4317S, RRID:AB_1903978) or antibody ab17722 (Abcam, # ab17722, RRID:AB_2278530) as described (Darnell et al., 2009).

### Hippocampal slice preparations

Transverse hippocampal slices (400 μm) were prepared as described (Lee et al., 2002). They were placed on the nylon mesh of an interface recording chamber (Fine Science Tools). Artificial CSF (ACSF) contained the following (in mm): 124 NaCl, 5 KCl, 1.6 MgCl_2_, 2 CaCl_2_, 26 NaHCO_3_, and 10 d-glucose. Slices were continuously perfused with ACSF bubbled with 95% O_2_/5% CO_2_ to maintain the pH near 7.4. The temperature was maintained at 33–35°C.

### Western blot analysis

Brains were collected and rinsed three times in PBS. They were homogenized in RIPA lysis buffer (Thermo Fisher Scientific) containing protease inhibitors (Roche) with a Dounce tissue homogenizer. Supernatants were collected after centrifugation (14,000 rpm for 15 min at 4°C), and protein concentrations were determined using the Bradford Protein Assay (Bio-Rad). Brain extracts (30 μg per well) were resolved by SDS-PAGE on 10% gradient polyacrylamide precast gels (Bio-Rad) and transferred to nitrocellulose membranes. Membranes were blocked for 1 h at room temperature with 5% nonfat dry milk (Bio-Rad) in Tris-buffered saline (TBS) with 0.01% Tween 20. Membranes were incubated overnight at 4°C with primary antibodies in blocking buffer. Primary antibodies were rabbit anti-FMRP (Abcam, 1:500 dilution) and mouse anti–γ-tubulin (Sigma-Aldrich, #T5326, RRID:AB_532292, 1:1000 dilution). Membranes were washed and incubated for 1 h with horseradish peroxidase–conjugated anti-rabbit and anti-mouse antibodies (Kindle Biosciences). Chemiluminescence levels were established using a Kwik Quant Imager (Kindle Biosciences). Bands were quantified using ImageJ software. FMRP levels were normalized to levels of γ-tubulin, which was used as a loading control.

### *In situ* hybridization

^35^S-labeled RNA probes directed against BC1 RNA were generated from plasmid pMK1 (Tiedge, 1991; Tiedge et al., 1991). The insert of this plasmid corresponds to the 60 3′-most nucleotides of rat BC1 RNA. 5′ BC1 sequences, which are homologous to repetitive ID elements (Iacoangeli and Tiedge, 2013; Eom et al., 2018), are thus avoided. We continue to rely on radioactive RNA probes, as nonradioactive BC RNA probes have in our hands resulted in inconsistent and artificial labeling. RNA probes were transcribed from pMK1 using T3 and T7 RNA polymerases, as described (Tiedge, 1991; Tiedge et al., 1991).

CGG and WT animals were perfusion-fixed with 4% formaldehyde (freshly prepared from paraformaldehyde) in PBS, brains were sectioned coronally at 10–12 μm, and specimens were postfixed by UV illumination (Tiedge, 1991). Tissue sections were hybridized with probes at 3–5 × 10^6^ cpm/μl in a solution containing 10 mm Tris/HCl, 0.6 m NaCl, 1 mm EDTA, 10 mm dithiothreitol, 0.1% bovine serum albumin, 0.02% Ficoll, 0.02% polyvinylpyrrolidone, 10 μg/ml salmon sperm DNA, 50 μg/ml yeast total RNA, 50 μg/ml *E. coli* transfer RNA, 50% formamide, 10% dextran sulfate, pH 7.5, at 50°C for 12–18 h. After hybridization, tissue sections were subjected to a wash in 4 liters of 2× SSC at 45°C for 1 h, an RNase digestion (30 mg/ml RNase A) in 10 mm Tris/HCl, 500 mm NaCl, pH 7.5, for 45 min at 37°C), a second wash in 4 liters of 2× SSC at 45°C for 1 h, and a high-stringency wash in 4 liters of 0.1× SSC, 0.05% sodium pyrophosphate, and 14 mm 2-mercaptoethanol for 3 h at 50°C, followed by an overnight wash in the same buffer at room temperature. Chemicals were from Sigma-Aldrich.

For data acquisition, we used a Microphot-FXA microscope (Nikon; Nikon instruments were purchased from Morrell Instruments; Muslimov et al., 2006, 2011, 2014). After emulsion autoradiography, tissue sections were imaged at room temperature using dark-field and bright-field optics. The following objectives were used: (1) Plan Fluor 10×/0.30, 160/0.17; (2) Ph2 Plan 20×/0.50, DL 160/0.17; (3) Ph3 DL Plan 40×/0.65, 160/0.17. Digital images were acquired with a Digital Sight DS-Fi1 5-megapixel charge-coupled device (CCD) camera (Nikon). Image analysis was performed using MetaMorph software (Molecular Devices); autoradiographic silver grain counts were performed by investigators not cognizant of animal genotypes. To establish RNA distribution profiles in hippocampal CA1, silver grain densities were measured across strata oriens, pyramidale, and radiatum at 50-μm-interval points, as described (Muslimov et al., 2004, 2006, 2011, 2014). Illustrations were generated using Photoshop and Illustrator software (Adobe Systems).

### Immunocytochemistry

Immunocytochemistry was performed with coronal brain sections prepared as described above (In situ hybridization). Antigen retrieval was performed by heating sections in citrate buffer (10 mm citric acid, 0.05% Tween 20, pH 6.0, adjusted using 1 N NaOH), in a water bath at 95°C for 30 min, after which sections were allowed to cool in the bath (by turning bath off) for 30 min. Sections were permeabilized using 0.2% Triton X-100 in PBS for 10 min at room temperature. Sections were subsequently incubated in superblock (0.01 m PBS, 0.05% Tween 20, 1% BSA, 1.5% normal goat serum, 1% sodium azide).

Primary antibodies (incubation in superblock overnight at 4°C) were as follows: anti-synaptophysin, monoclonal, raised in rabbit (LifeSpan, #LS-C210604, RRID:AB_2722673, dilution 1:500), anti-MAP2, monoclonal, raised in mouse (Abcam #ab28032, RRID:AB_776173, dilution 1:1000).

The next morning, sections were washed four times in 0.01 m PBS with 0.05% Tween 20 (10 min each, room temperature) and incubated with species-specific secondary antibodies in 0.01 m PBS with 0.05% Tween 20, 1% BSA, 1.5% normal goat serum, and 1% sodium azide for 2 h at room temperature. Secondary antibodies were used as follows: anti-rabbit labeled with Alexa Fluor 488 (Thermo Fisher Scientific #A-11008, RRID:AB_143165, dilution 1:500) for synaptophysin-labeled sections, and anti-mouse labeled with Alexa Fluor 594 (Thermo Fisher Scientific #R37121, RRID:AB_2556549, dilution 1:250) for MAP2-labeled sections. Background labeling was ascertained by performing experiments in the absence of primary antibodies.

Microscopy was performed on a Microphot-FXA microscope (Nikon; Eom et al., 2014). The following objectives were used: (1) Plan Fluor 10×/0.30, 160/0.17; (2) Ph2 Plan 20×/0.50, DL 160/0.17; (3) Ph3 DL Plan 40×/0.65, 160/0.17. Digital images were acquired with a Digital Sight DS-Fi1 5-megapixel CCD camera (Nikon). Fluorescence intensities were quantified using ImageJ (NIH).

### Intracellular recordings

Hippocampal slices were allowed to recover from the isolation procedure for at least 1.5 h. Intracellular recordings were performed in CA3 pyramidal cells using an Axoclamp 2A amplifier (Molecular Devices). Electrodes were pulled with thin-walled glass tubing (World Precision Instruments) and had resistances of 30–50 MΩ when filled with potassium acetate (2 m). Voltage signals were displayed on an oscilloscope (DSO 400; Gould Instruments) and digitized and stored on an Intel Pentium-based computer using a Digidata 1322A converter controlled by pClamp 8 software (Molecular Devices).

Pharmacological agents were used as follows. Baseline epileptiform activities for experiments in WT- and CGG-animal hippocampal slices were elicited by continuous bath perfusion of the GABA_A_ antagonist bicuculline (50 μm). The NMDAR antagonist (*RS*)-CPP (20 μm) was added to the perfusate of those hippocampal slice preparations that did not transition from short bursts to prolonged bursts within 60 min of bicuculline application. The group I metabotropic glutamate receptor 1 (mGluR1)-selective antagonist (*S*)-(+)-α-amino-4-carboxy-2-methylbenzeneacetic acid (LY367385, 80 μm) and the mGluR5-selective antagonist 2-methyl-6-(phenylethynyl)-pyridine hydrochloride (MPEP, 80 μm) were used to test mGluR dependence of epileptiform activities (obtained from Tocris Bioscience).

Data analysis was performed as follows. Durations of individual synchronized discharges were measured from the beginning of the first action potential to the repolarization of the last action potential of the discharge. Membrane potentials were kept within a few millivolts throughout the experiment. Frequency histograms included the durations of all synchronized discharges that were recorded in 6-min periods for each slice under the respective experimental conditions. Based on the distribution of the synchronized discharge durations reported previously (Chuang et al., 2005; Fig. 1*Dbi*), “short” and “long” bursts refer to events shorter and longer, respectively, than 1.5 s. Clampfit (Molecular Devices) and SigmaPlot (SPSS) software was used for data analysis.

### Audiogenic seizures

Audiogenic seizures were induced as follows (Zhong et al., 2009, 2010). 19–21-d-old mice were placed in a plastic cage which contained, mounted into the top, a personal alarm device (TBO-Tech). Convulsive seizures were recorded during an auditory stimulation (120 dB) period of 15 min.

To examine whether audiogenic seizure induction required de novo protein synthesis, CGG mice were injected i.p. with 75 mg/kg anisomycin 1 h before auditory stimulation. To examine dependence of group I mGluR signaling, CGG mice were injected i.p. with 40 mg/kg mGluR5 antagonist MPEP (Bio-Techne) 30 min before auditory stimulation. Doses of anisomycin and MPEP used here were the same as previously reported with BC1 knockout (KO) mice (Zhong et al., 2009).

### Self-grooming

Animals were scored for self-grooming behavior as described (McFarlane et al., 2008; Iacoangeli et al., 2017). For habituation, an animal was kept in an empty box without bedding for 10 min. The cumulative time a mouse spent self-grooming was then stopwatch-recorded by an investigator (positioned at a distance of 2 m) for 10 min.

### Attentional Set Shift Task

The Attentional Set Shift Task (ASST) protocol was carried out as recently described for BC1 KO animals (Iacoangeli et al., 2017). It has been modified from previous work (Colacicco et al., 2002; Garner et al., 2006; Scheggia et al., 2014; Tait et al., 2014) as follows.

A Plexiglas apparatus was used in which a crosswise moveable divider gate separated the holding area from the testing area, the latter equipped with an immovable central divider that split the area lengthwise into two compartments. In initial shaping sessions, animals were trained to retrieve a food reward (a quarter piece of honey-nut Cheerio–type cereal) from two clay pots in the testing area, one on either side of the central divider. Animals were allowed to retrieve rewards from bowls that were initially free of scent, digging medium, or excess texture. In the following shaping session, the bowls were filled with shredded tissue as a digging medium, and the rewards were placed on top of the medium. In the final shaping session, the rewards were buried in the digging medium.

In discrimination training sessions, animals had to learn to use up to three classes of discriminanda (dimensions) in their reward retrieval strategies: the textures of the bowls’ outer surfaces, the digging media in which the rewards were buried, and the scents associated with the digging media and thus the bowls. In four phases over a 3-d period, animals engaged in a total of nine learning sessions that included Simple Discrimination (SD) Learning, Compound Discrimination (CD) Learning, and Conflict Learning sessions (Table 1). Phase 1 began with an initial Simple Discrimination Learning session, sometimes considered part of habituation/shaping training (Cao et al., 2012), in which animals learned that a specific dimension (e.g., odor) was reward-relevant. The bowl associated with the reward-predictive scent was baited with the food reward, hidden in the digging medium. The other bowl (nonpredictive scent) was not baited. In a subsequent Compound Discrimination Learning session, two additional stimuli were introduced as “distractors” (Jazbec et al., 2007) in the reward-irrelevant dimensions digging medium and bowl texture (Table 1). In the final Conflict Learning session of Phase 1, a stimulus in the reward-relevant dimension (e.g., odor) was switched from reward-predictive to nonpredictive and vice versa. In Phases 2 and 3, reward-predictive and nonpredictive stimuli changed, but odor was kept as the reward-relevant dimension (intradimensional shift, IDS). In Phase 4, the reward-relevant dimension changed from odor to digging medium (extradimensional shift, EDS).

**Table 1.** The nine learning sessions of the ASST protocol

Session	Phase	Day	Dimension	Stimulus pairing	
SD Learning	1	1			
			Odor	Sage*	Cinnamon
			Medium	Aspen bedding	Aspen bedding
			Texture	Plastic wrap	Plastic wrap
CD Learning 1	1	2		
			Odor	Sage*	Cinnamon
			Medium	Aspen bedding	Moss
			Texture	Plastic wrap	Bubble wrap
Conflict Learning 1	1	2		
			Odor	Sage	Cinnamon*
			Medium	Aspen bedding	Moss
			Texture	Plastic wrap	Bubble wrap
CD Learning 2 (IDS)	2	2			
			Odor	Cumin*	Rosemary
			Medium	Gravel	Pellets
			Texture	Wax paper	Aluminum foil
Conflict Learning 2 (IDS)	2	2			
			Odor	Cumin	Rosemary*
			Medium	Gravel	Pellets
			Texture	Wax paper	Aluminum foil
CD Learning 3 (IDS)	3	3			
			Odor	Oregano*	Nutmeg
			Medium	Packing peanuts	Shredded paper
			Texture	Smooth cardboard	Cloth
Conflict Learning 3 (IDS)	3	3			
			Odor	Oregano	Nutmeg*
			Medium	Packing peanuts	Shredded paper
			Texture	Smooth cardboard	Cloth
CD Learning 4 (EDS)	4	3			
			Odor	Thyme	Cloves
			Medium	Perlite*	Sand
			Texture	Fine sandpaper	Coarse sandpaper
Conflict Learning 4 (EDS)	4	3			
			Odor	Thyme	Cloves
			Medium	Perlite	Sand*
			Texture	Fine sandpaper	Coarse sandpaper

*Reward predictive stimulus.

CD, compound discrimination; SD, simple discrimination. Intradimensional shift (IDS) was applied in Phases 2 and 3, extradimensional shift (EDS) in Phase 4. Adopted from Iacoangeli et al. (2017).

Animal performance was scored as follows. For a successful completion of a training session, i.e., to reach criterion, an animal had to make a minimum of 8 correct choices in 10 consecutive trials. Scored were (a) the number of incorrect choices (errors to criterion, ETC) and (b) the number of trials needed to complete the session (trials to criterion, TTC). An animal that did not reach criterion was excluded from analysis.

If significant differences between animal groups were detected in Conflict Learning sessions, the experimental results were analyzed for the types of error committed (Baker et al., 2011; Amodeo et al., 2012). We differentiated two types of error, perseverative and regressive. In the former case, an animal in a Conflict Learning session continued to adhere to a choice that was incorrect but had been correct in the preceding CD session. In the latter case, an animal in a Conflict Learning session would make one or more incorrect choices even if it had made at least one correct choice earlier in the same session.

### Spontaneous alternation

Spontaneous alternation was analyzed in T-maze experiments (Deacon and Rawlins, 2006; Iacoangeli et al., 2017). An animal was placed in the start arm, and once it had chosen and entered a goal arm, the gate of that arm was lowered and the animal allowed to remain in place for 30 s. The animal was retrieved and placed back in the start arm, allowing it to make a second choice. Spontaneous alternation was recorded and scored as percentage of total goal arm entrances. 10 trials were performed per animal, with an intertrial interval of 20 minutes (Deacon et al., 2003; Iacoangeli et al., 2017).

### Statistical analyses

SPSS Statistics (IBM), SAS (version 9.4, SAS Institute Inc.), and Statistica (StatSoft) software were used for statistical analyses. Adobe Illustrator (Adobe Systems) and Prism (GraphPad Software) was used to generate graphs.

The Cox proportional hazards regression model was used to analyze ASST data (Jahn-Eimermacher et al., 2011; Iacoangeli et al., 2017). The predictor variable genotype (CGG vs. WT) was used in comparing the number of ETC and TTC. ETC and TTC data were examined stratified for the type of learning session, using the robust variance estimator (Lin and Wei, 1989) to account for the correlated observations (ETC and TTC values) for each animal. Hazard ratios (HRs) were examined to compare performance scores between groups (Iacoangeli et al., 2017). For example, an HR <1 is an indication of poorer performance of the first group, with higher ETC and TTC than the second group. The Cox model was chosen over ANOVA because of the skewed distribution of the outcomes and the advantages of the former approach compared to the latter (Jahn-Eimermacher et al., 2011).

One-way ANOVA (with Dunnett’s *post hoc* analysis) was used to examine RNA localization data. One-way ANOVA (with *post hoc* Tukey HSD test) was used to analyze intracellular recordings data (Chuang et al., 2005; Zhong et al., 2009). Audiogenic seizure data were analyzed using Fisher’s exact test (Zhong et al., 2009). The Mann–Whitney test was used for self-grooming, T-maze, and type of error data analysis (Iacoangeli et al., 2017).

Results of statistical analyses are reported in the main text or in figure legends, as appropriate. Data in the figures are displayed as mean ± SEM unless noted otherwise. Levels of significance are indicated in the figures as follows: *, *p* < 0.05; **, *p* < 0.01; ***, *p* < 0.001.

## Results

### Experimental design

Molecular-cellular, physiological, and cognitive-behavioral approaches were employed to test the key predictions of our working hypothesis. Fundamental to this hypothesis is the concept of CGG-repeat–BC RNA competition; therefore, this concept was addressed first. At the next level, we tested the prediction that such competition will cause mislocalization of BC1 RNA in vivo, working with CGG-repeat KI animals. In the CGG-repeat KI animal model, endogenous murine (CGG)_8_ repeats were replaced with human (CGG)_180_ repeats (Brouwer et al., 2008). These mice are in the following referred to as (CGG)_180_ or simply CGG animals. CGG-repeat lengths and brain FMRP levels of such animals were monitored as described in Methods. At the third level, we tested the prediction that phenotypic features caused by lack of BC1 RNA, i.e., seizure activity and cognitive impairment (Zhong et al., 2009; Iacoangeli et al., 2017), are recapitulated in CGG animals with significantly reduced synapto-dendritic presence of the RNA.

### CGG-repeat competition with BC RNAs for hnRNP A2

The group of regulatory BC RNAs includes primate BC200 RNA and rodent BC1 RNA (Iacoangeli and Tiedge, 2013; Eom et al., 2018). Both RNAs are expressed in neurons, localize to synapto-dendritic domains, and participate in activity-dependent translational control mechanisms at the synapse (Eom et al., 2011, 2014; 2018; Muslimov et al., 2011; Iacoangeli and Tiedge, 2013). The genes encoding these RNAs are not evolutionarily orthologous, as they originated, by retroposition, via distinct phylogenetic pathways (Martignetti and Brosius, 1993a, b; Iacoangeli and Tiedge, 2013; Eom et al., 2018). Primate BC200 RNA and rodent BC1 RNA therefore operate as functional analogs rather than as phylogenetic orthologs (Iacoangeli and Tiedge, 2013; Eom et al., 2018). To establish primate–rodent functional analogy with respect to CGG repeat competition, we examined CGG-repeat competition with BC200 RNA and BC1 RNA in parallel.

**Figure 2.**
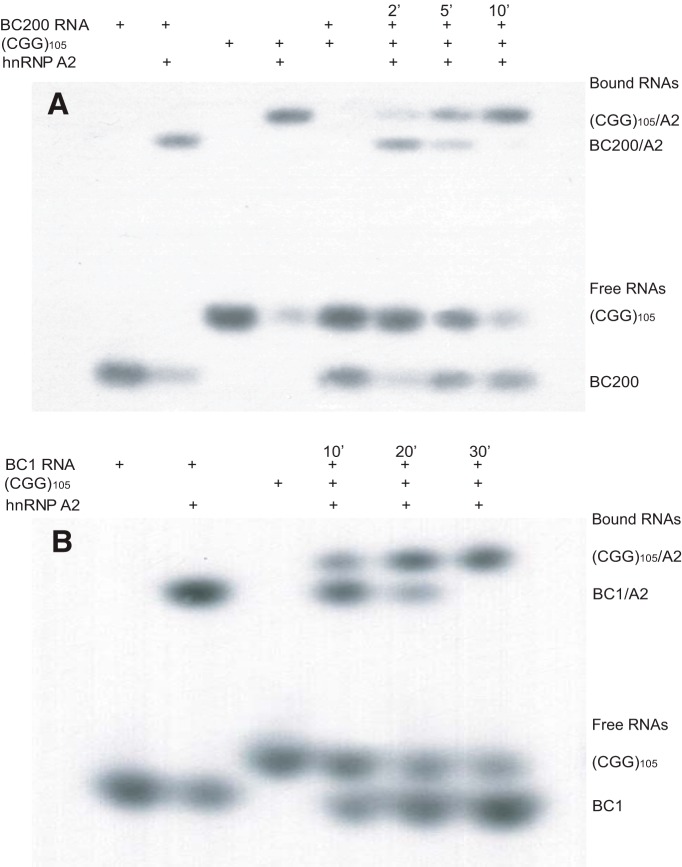
Competition of (CGG)_105_ RNA with primate BC200 RNA and rodent BC1 RNA for binding to hnRNP A2. EMSA competition analysis examined binding of human BC200 RNA (***A***) and rat BC1 RNA (***B***) to RNA transport factor hnRNP A2. ***A***, Gel was loaded with BC200 RNA (BC200), (CGG)_105_ RNA [(CGG)_105_], and hnRNP A2 (A2) as follows: 1, BC200; 2, BC200 + A2; 3, (CGG)_105_; 4, (CGG)_105_ + A2; 5, BC200 + (CGG)_105_; 6–8, BC200 + A2 + (CGG)_105_ at 2, 5, or 10 min of incubation. ***B***, Gel was loaded with BC1 RNA (BC1), (CGG)_105_, and A2 as follows: 1, BC1; 2, BC1 + A2; 3, (CGG)_105_; 4–6, BC1 + A2 + (CGG)_105_ at 10, 20, or 30 min of incubation time. Components were used at 100 nm (Muslimov et al., 2011) except for (CGG)_105_ RNA, which was used at a 1:3 molar ratio to the respective BC RNA.

We used electrophoretic mobility shift assay (EMSA) analysis (Muslimov et al., 2006, 2011, 2014) to ascertain molecular competition between BC200 RNA and BC1 RNA, on one hand, and CGG repeat RNA on the other, for RNA transport factor hnRNP A2 (Fig. 2). Fig. 2*A* shows that interactions of human BC200 RNA with recombinant hnRNP A2 caused a significant reduction in electrophoretic mobility of the radiolabeled RNA. Interaction of (CGG)_105_ repeat RNA with hnRNP A2 resulted in an analogous but non-identical shift to lower mobility. We next pre-incubated hnRNP A2 with BC200 RNA for 15 min. After addition of (CGG)_105_ repeat RNA (molar ratio to BC200 RNA 1:3), we observed a complete displacement of BC200 RNA from hnRNP A2 in less than 10 min (Fig. 2*A*). Over the same time period, levels of bound (CGG)_105_ repeat RNA and free BC200 RNA increased, while levels of free (CGG)_105_ repeat RNA decreased.

Analogous results were obtained when rat BC1 RNA was used in EMSA competition assays (Fig. 2*B*). We pre-incubated hnRNP A2 with BC1 RNA for 15 min, at which time point (CGG)_105_ repeat RNA was added at a 1:3 molar ratio to BC1 RNA. BC1 RNA was completely displaced from hnRNP A2 within 30 min (Fig. 2*B*). Over the same period of time, levels of bound (CGG)_105_ repeat RNA and free BC1 RNA increased, while levels of free (CGG)_105_ repeat RNA decreased, a result analogous to the one obtained with BC200 RNA (Fig. 2*A*). We conclude that (CGG)_105_ repeat RNA effectively competes both primate BC200 RNA and rodent BC1 RNA off hnRNP A2 within minutes. This displacement appears to occur more rapidly with BC200 RNA than with BC1 RNA, possibly reflecting a lower BC RNA–hnRNP A2 dissociation rate constant in the latter case.

### Mislocalization of BC1 RNA in CGG animal brains

Using a microinjection approach with sympathetic neurons in primary culture, we have previously reported that interactions of BC1 RNA with hnRNP A2 are required for dendritic delivery (Muslimov et al., 2006, 2011). Given that BC1 RNA is effectively displaced from hnRNP A2 by (CGG)_105_ repeat RNA in vitro (Fig. 2*B*), we asked whether such displacement would cause dendritic localization impairments of BC1 RNA in vivo.

We used (CGG)_180_ animals to address this question. CGG-repeat lengths in experimental animals were verified as shown in Fig. 1, confirming that Fmr1 mRNA in these animals carried 180 (or close to 180) CGG repeat units. We maintained that endogenous Fmr1 mRNA with 180 CGG repeat units would be at least as effective in displacing BC1 RNA from hnRNP A2 as an RNA with 105 CGG repeat units applied in vitro at equivalent concentrations (see Methods and Muslimov et al., 2011).

**Figure 3.**
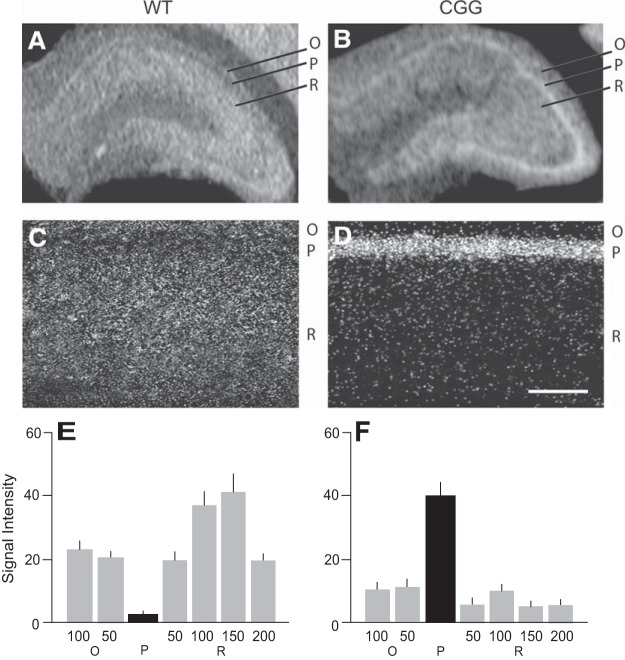
Impaired dendritic localization of BC1 RNA in (CGG)_180_ animals. CA1 strata oriens (O), pyramidale (P), radiatum (R) are indicated. ***A***, ***B***, Coronal sections through hippocampal regions (film autoradiograms) reveal that BC1 RNA (white signal) is concentrated in CA1 strata oriens and radiatum (i.e., dendritic layers) of WT animals but, in contrast, is concentrated in stratum pyramidale (i.e., cell body layer) of CGG animals. ***C***, ***D***, Emulsion autoradiography of the CA1 hippocampal region confirms predominantly dendritic BC1 labeling (white signal) in WT CA1 but predominantly somatic labeling in CGG CA1. Scale bar for ***C*** and ***D***: 100 μm. ***E***, ***F***, Quantitative analysis of emulsion autoradiographs: one-way ANOVA, *p* < 0.001. Dunnett’s *post hoc* analysis, comparison of signal intensities (given in relative units) in stratum P (center), in stratum O at distances of 50 and 100 μm from edge of stratum P, and in stratum R at distances of 50, 100, 150, and 200 μm from edge of stratum P, between WT and CGG animals: *p* < 0.001 for all sample points. *n* = 4 for WT and CGG animals. Error bars indicate SEM.

We performed in situ hybridization (Lin et al., 2001) with brains from 12-wk-old CGG animals (Fig. 3). Strain-, sex-, and age-matched WT (see also Methods) mice were used in parallel. Fig. 3*A* shows that in WT mouse hippocampus, comparatively high BC1 RNA signal intensities are detectable in synapto-dendritic strata oriens and radiatum of CA1, with lower signal intensities in somatic statum pyramidale of the same region. The results indicate that BC1 RNA is enriched in the basal (oriens) and apical (radiatum) dendritic arborizations of WT mouse CA1 pyramidal cells, in agreement with previous work with rats (Lin et al., 2001). In CGG animal brains, in contrast, in situ hybridization revealed a strikingly different somato-dendritic distribution of BC1 RNA in CA1: here, BC1 RNA signal intensities were high in CA1 somatic stratum pyramidale but low in CA1 dendritic strata oriens and radiatum (Fig. 3*B*).

The above results were substantiated by higher-magnification light-microscopic analysis following emulsion autoradiography (Fig. 3*C*, *D*). Again, BC1 RNA labeling was high in WT strata oriens and radiatum but lower in WT stratum pyramidale (Fig. 3*C*) whereas, conversely, labeling was low to undetectable in CGG animal strata oriens and radiatum but very strong in stratum pyramidale (Fig. 3*D*). We conclude that in neurons expressing (CGG)_180_ Fmr1 mRNA, the dendritic localization of BC1 RNA is severely reduced, while somatic retention is significantly increased (see quantitative analysis in Fig. 3*E* and *F*).

Phenotypic alterations in BC1 KO animals (Zhong et al., 2009; Briz et al., 2017; Iacoangeli et al., 2017) are caused by global lack of regulatory BC1 RNA. It was formally possible that phenotypic alterations in CGG animals were also caused by a reduction of brain BC1 RNA expression levels, in addition to cellular BC1 RNA mislocalization as described above. To address this question, we used in situ hybridization to establish brain levels of BC1 RNA in CCG animals versus matched WT animals. No significant differences were detected in BC1 RNA brain expression levels between CGG and WT animals (one-way ANOVA, *p* = 0.94, 4 animals with 11 brain sections per group; not illustrated). The data indicate that BC1 RNA expression levels are unaltered in CGG animal brains.

**Figure 4.**
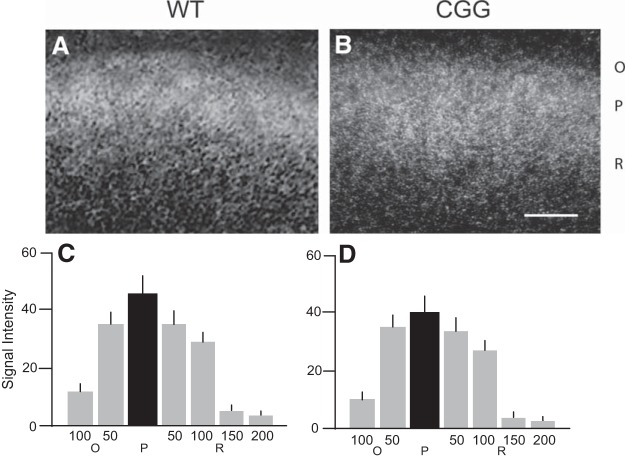
Somato-dendritic distribution of MAP2 mRNA in WT and CGG brains. ***A***, ***B***, MAP2 mRNA distribution in hippocampal CA1 of WT and CGG animals. CA1 strata oriens (O), pyramidale (P), radiatum (R) are indicated. Scale bars, 100 μm. ***C***, ***D***, Quantitative analysis: one-way ANOVA, *p* = 0.94065. Comparison of signal intensities (given in relative units) in stratum P (center), in stratum O at distances of 50 and 100 μm from edge of stratum P, and in stratum R at distances of 50, 100, 150, and 200 μm from edge of stratum P, between WT and CGG animals: *p* > 0.05 for all sample points. *n* = 4 for WT and GCC animals.

The obtained results raise the question whether reduced dendritic localization in the presence of (CGG)_180_ Fmr1 mRNA is a feature specific to regulatory BC1 RNA or is rather a general feature that can also be observed with dendritic mRNAs. To address this question, we performed in situ hybridization directed at dendritic MAP2 mRNA (Garner et al., 1988; Paradies and Steward, 1997). We observed no significant differences in labeling intensities and distribution between CGG and corresponding WT animal brains (Fig. 4). In CA1, MAP2 mRNA labeling distribution in strata pyramidale, oriens, and radiatum was indistinguishable between CGG and WT animal CA1 fields (Fig. 4*A*, *B*): in both cases, we observed the typical, previously reported (Garner et al., 1988; Paradies and Steward, 1997), MAP2 mRNA labeling pattern. The data indicate that the somato-dendritic distribution of MAP2 mRNA is unaltered in CGG animal pyramidal neurons, in comparison with WT pyramidal neurons.

### Phenotypic alterations

The data shown in Fig. 3 indicate that in neurons expressing (CGG)_180_ Fmr1 mRNA, the subcellular localization of endogenous BC1 RNA is severely compromised, as it is barely detectable in synapto-dendritic regions where it is abundant in WT neurons. Lack of BC1 RNA causes neuronal hyperexcitability and cognitive impairment (Zhong et al., 2009, 2010; Chung et al., 2017; Iacoangeli et al., 2017). Our working hypothesis predicted that significantly diminished levels of BC1 RNA at synapto-dendritic sites of function would find expression in analogous phenotypic alterations. We worked with young-adult (CGG)_180_ animals to experimentally test this prediction.

#### Neuronal hyperexcitability: prolonged epileptiform discharges

Lack of BC1 RNA causes neuronal hyperexcitability that is detected in vitro as prolonged discharges in hippocampal slices and in vivo in the form of audiogenic seizures (Zhong et al., 2009, 2010). To test the prediction that such hyperexcitability manifests in CGG animal cortical circuits, we performed intracellular recordings from CA3 pyramidal cells in hippocampal slice preparations after synaptic activation of group I mGluRs (Chuang et al., 2005; Zhong et al., 2009). Synaptic release of glutamate was induced by application of the GABA_A_ receptor antagonist bicuculline, and the functional consequences of such activation were recorded as synchronized discharges from CA3 glutamatergic principal neurons (Chuang et al., 2005).

**Figure 5.**
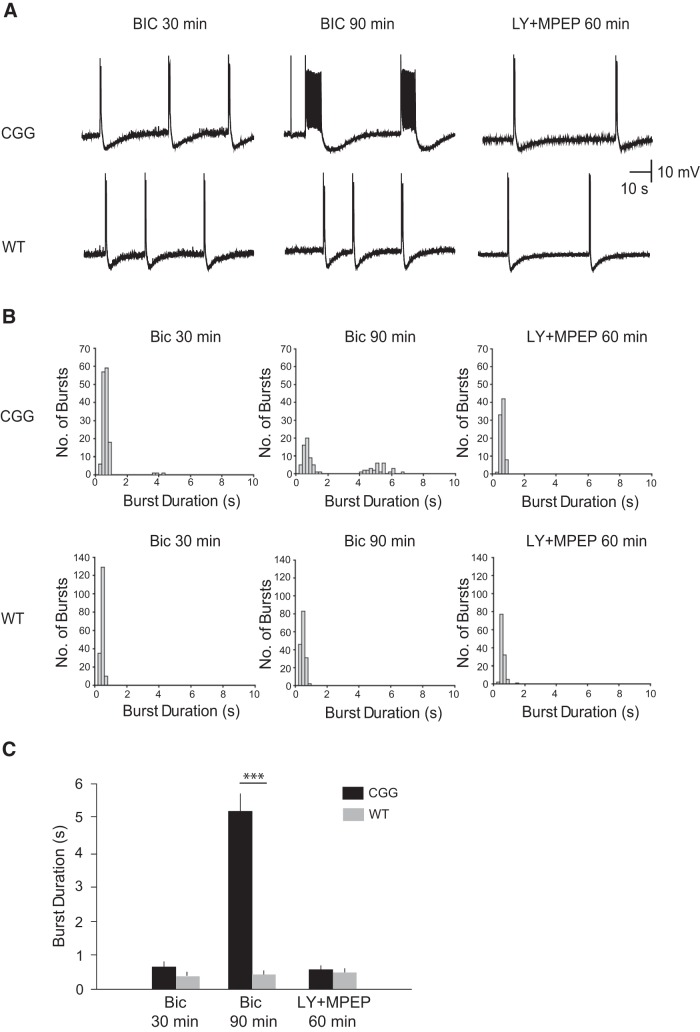
Prolonged epileptiform discharges in CGG CA3 pyramidal cells. The GABA_A_ receptor antagonist bicuculline induced group I mGluR-mediated prolonged epileptiform discharges in CA3 hippocampal pyramidal cells. ***A***, Intracellular recordings of the spontaneous activity of a CA3 pyramidal cell in a CGG hippocampal slice preparation (upper panels) and of a CA3 pyramidal cell in a WT hippocampal slice preparation (lower panels) after perfusion with bicuculline (50 μm). Within 30 min after addition of bicuculline, short synchronized discharges were elicited in both preparations (left). Continuous application of bicuculline induced prolonged synchronized discharges (4–7 s) in CGG animal preparations but not in WT animal preparations (middle). Addition of group I mGluRs antagonists LY367385 and MPEP (80 μm) reversed prolongation of synchronized discharges in CGG animals (right). ***B***, Frequency histograms of all synchronized bursts recorded during a 6-min period of stable rhythmic activity at three time points: Bic 30 min; Bic 90 min; LY367385 + MPEP 60 min. ***C***, Summary bar graph of average burst durations in CGG and WT preparations. The average burst duration in CGG preparations at Bic 90 min was significantly higher than that observed in WT preparations (5.106 ± 0.148 s vs. 0.504 ± 0.01 s; one-way ANOVA, *post hoc* Tukey HSD test, *p* < 0.001).

In hippocampal slice preparations from 8 CGG animals, intracellular recordings detected short rhythmic synchronized discharges (average duration: 0.686 ± 0.042 s) 30 min after application of bicuculline (Fig. 5*A*, *B*, upper panels). Over the course of the next 60 min, in preparations from 6 of 8 CGG animals, short synchronized discharges induced mGluR-mediated responses which extended discharge durations, causing the emergence of prolonged synchronized discharges. After a total of 90 min of bicuculline perfusion, these 6 CGG animal preparations displayed prolonged epileptiform discharges with an average duration of 5.105 ± 0.148 s (Fig. 5*A*, *B*, upper panels).

Addition of the respective mGluR1 and mGluR5 blockers LY367385 and MPEP resulted in a complete reversion of prolonged to short discharges (average duration: 0.640 ± 0.011 s) within 60 min (Fig. 5*A*, *B*, upper panels). A histogram plot (Fig. 5*B*, upper panels) reveals two populations of synchronized discharge durations after 90 min of perfusion with bicuculline but only one population after 60 min of perfusion with group I mGluRs blockers. We note that in BC1 KO animals, prolonged discharges of an average duration of 5.475 ± 0.124 s were observed following perfusion with bicuculline, and that such prolonged discharges were abolished by mGluR1 and mGluR5 blockers LY367385 and MPEP (Zhong et al., 2009).

In clear contrast to CGG-animal preparations, WT-animal preparations never displayed prolongation of synchronized discharges (Fig. 5*A*, *B*, lower panels; *n* = 6). After application of bicuculline, average burst durations remained stable at <1.5 s, and application of group I mGluR blockers had no effect on the duration of synchronized discharges (Fig. 5*A*, *B*, lower panels: Bic 30 min: 0.475 ± 0.006 s; Bic 90 min: 0.504 ± 0.01 s; LY367385 and MPEP 60 min: 0.586 ± 0.013 s).

The combined data of Fig. 4 reveal neuronal hyperexcitability in CGG-animal CA3 cells in which dendritic localization of BC1 RNA is diminished (Fig. 3). Such hyperexcitability is similar to that in hippocampal preparations from BC1 KO animals (Zhong et al., 2009), as the transition from short to prolonged synchronized bursts is analogous.

#### Neuronal hyperexcitability: seizure activity

**Figure 6.**
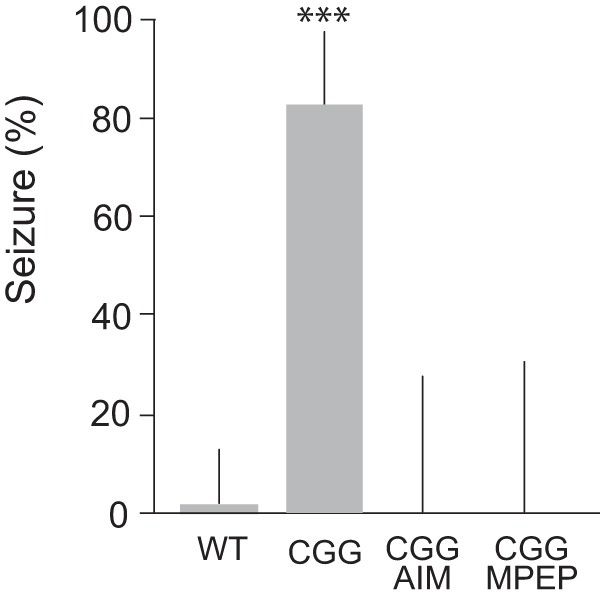
Audiogenic seizures in CGG animals. Significantly increased propensity for audiogenic seizures was observed with CGG mice (*n* = 42), in comparison with WT mice (*n* = 30). Seizures were not observed in CGG mice injected with anisomycin (75 mg/kg i.p.; *n* = 12) or MPEP (40 mg/kg i.p.; *n* = 10). Fisher’s exact test, *p* < 0.001. Error bars represent 95% confidence intervals.

In neural networks, hyperexcitability manifests in epileptogenic susceptibility that, in rodents in vivo, can be diagnosed as a propensity for sound-induced (audiogenic) seizure activity. In the absence of BC1 RNA, epileptic activity following auditory stimulation takes the form of generalized, tonic-clonic seizures (Zhong et al., 2009, 2010). We now observe similar audiogenic seizures with CGG animals (Fig. 6).

Upon auditory stimulation (120 dB), CGG animals within seconds initiated wild running and jumping, activity that was followed (within 1 min) by clonic-tonic convulsions. 83% of CGG animals underwent such seizures, in comparison with 84% of BC1 KO animals (Zhong et al., 2009). The age of the animals in both groups was 18–21 days. Audiogenic seizures were not observed upon auditory stimulation of CGG animals that had been injected with (a) mGluR5 antagonist MPEP or (b) protein synthesis inhibitor anisomycin (Fig. 6). Analogous dependence of audiogenic seizures on group I mGluR activation and de novo protein synthesis has been observed with BC1 KO animals (Zhong et al., 2009). The combined results support the notion that seizure susceptibility in young CGG mice and young BC1 KO mice can be attributed to a common molecular-cellular shortcoming: lack or diminished presence of translational regulator BC1 RNA in synapto-dendritic domains. The fact that BC1 RNA acts as a break on group I mGluR-stimulated protein synthesis (Zhong et al., 2009; Iacoangeli and Tiedge, 2013; Eom et al., 2018) explains the requirement for group I mGluR signaling and protein synthesis in the manifestation of neuronal hyperexcitability in cases when BC1 RNA control is impaired.

#### Cognitive abnormalities

Impaired cognitive competence has recently been reported for animals lacking regulatory BC1 RNA (Chung et al., 2017; Iacoangeli et al., 2017). Our working hypothesis predicts that such phenotypic deficit caused by the absence of BC1 RNA will be recapitulated in animals with severely reduced synapto-dendritic expression of the RNA. In a final test of this prediction, we examined cognitive performance of CGG animals.

##### Self-grooming.

**Figure 7.**
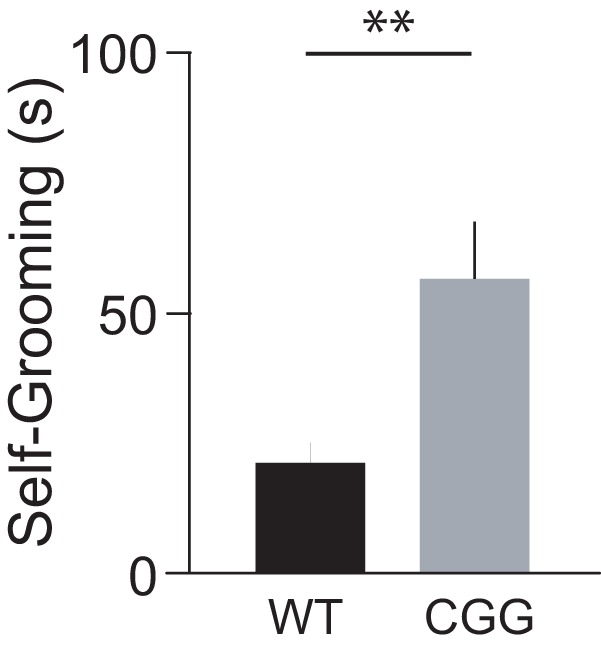
Self-grooming of CGG animals. CGG mice spent significantly more time self-grooming than WT mice (Mann–Whitney test, *p* = 0.004). *n* = 13 for each group.

Repetitive, excessive self-grooming has been associated with autism-like deficits in rodents (McFarlane et al., 2008; Silverman et al., 2010; Lai et al., 2014). BC1 KO animals have recently been shown to exhibit this type of behavioral impairment (Iacoangeli et al., 2017). Here we examined (CGG)_180_ animals for such alterations. In a self-grooming test, the cumulative time an animal spent in all-body grooming activity was recorded over a 10-min time window. We found that CGG mice spent significantly more time self-grooming than WT mice (Fig. 7).

##### Cognitive flexibility.

Impaired cognitive flexibility has been reported for animals lacking BC1 RNA (Chung et al., 2017; Iacoangeli et al., 2017). Here we the used the ASST (Birrell and Brown, 2000; Colacicco et al., 2002; Garner et al., 2006; Iacoangeli et al., 2017) to examine cognitive flexibility of CGG animals. Animals learn to retrieve a food reward (cereal) from one of two bowls on the basis of one of three types of cues (known as dimensions): outer texture of the bowl, nature of the digging medium, or scent of the medium (see Methods; see also Table 1 and Iacoangeli et al., 2017). We recorded (a) the number of errors that an animal committed, by choosing the bowl without reward, before reaching criterion (errors to criterion, ETC) and (b) the number of trials needed to reach criterion (trials to criterion, TTC). Criterion was defined as making at least 8 correct choices in 10 consecutive trials. In the first three phases of ASST analysis, odor was used as the reward-relevant dimension, whereas the nature of the digging medium was reward-relevant in the fourth phase (Iacoangeli et al., 2017).

##### Phase 1.

**Figure 8.**
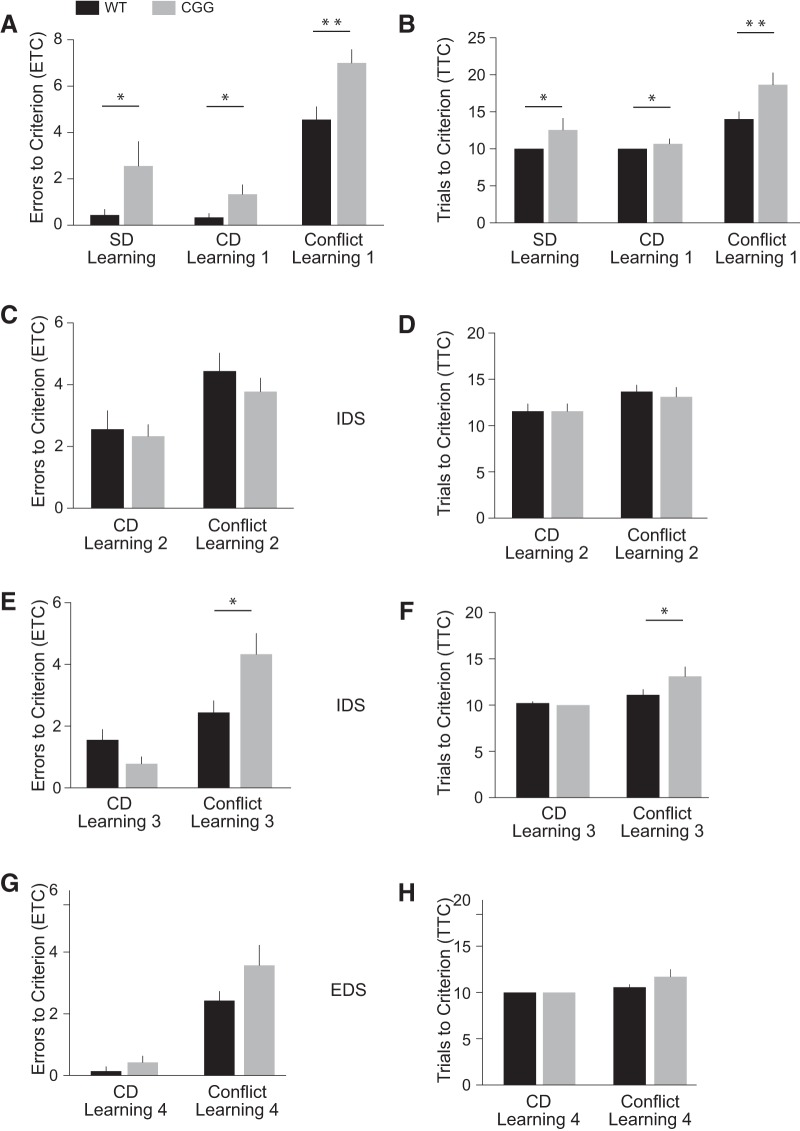
Cognitive flexibility is impaired in CGG animals. Numbers of ETC (***A***, ***C***, ***E***, and ***G***) and TTC (***B***, ***D***, ***F***, and ***H***) were recorded. ***A***, ***B***, In Phase 1, CGG animals were significantly impaired, in comparison to WT mice, in sessions SD learning, CD Learning, and Conflict Learning. ***C***, ***D***, CGG animals and WT animals performed similarly in Phase 2 sessions CD Learning 2 and Conflict Learning 2. ***E***, ***F***, In Phase 3, CGG and WT animals performed comparably in session CD Learning 3 but CGG animals displayed continued cognitive impairment in session Conflict Learning 3. ***G***, ***H***, In Phase 4, no significant differences were observed in the performances of CGG and WT animals in session CD Learning 4 and Conflict Learning 4. EDS, extradimensional shift; IDS, intradimensional shift. *n* = 9 for each animal group (CGG and WT).

The odor pairing in the initial Simple Discrimination (SD) Learning session was sage (reward-predictive) and cinnamon. Additional stimulus pairings (i.e. digging media and bowl textures; see Methods, Table 1) were used as distractors and had to be disregarded by the animals as reward-irrelevant dimensions. The initial SD Learning session was followed by a Compound Discrimination (CD) Learning session in which additional novel stimuli were included in the two reward-irrelevant dimensions (Table 1). CGG mice were impaired in both the SD and CD Learning sessions (Fig. 8*A*, *B*). Compared with WT mice, CGG mice committed a significantly higher number of ETC (Fig. 8*A*) and consequently needed increased numbers of trials (TTC, Fig. 8*B*) to complete the SD Learning session (HR = 0.45, 95% CI 0.23–0.92, *p* = 0.027) and the CD Learning session (HR = 0.64, 95% CI 0.45–0.92, *p* = 0.015). Initial discrimination learning was also found to be impaired in BC1 KO animals (Iacoangeli et al., 2017) and is sometimes considered an extension of the habituation phase (Cao et al., 2012).

In the subsequent Conflict Learning session, stimulus pairings remained unchanged except that cinnamon rather than sage was now the reward-predictive odor. The performance of CGG animals was significantly impaired in this session, as indicated by the higher number of ETC and TTC in comparison with WT mice (Fig. 8*A*, *B*; HR = 0.33, 95% CI 0.14–0.76, *p* = 0.009). The results reveal impaired cognition in CGG animals: when presented with changed external contingencies, they continued to apply a previously adopted strategy that had become inappropriate in the new situational context.

##### Phase 2.

All stimuli were changed in Phase 2 of the ASST analysis, with odor remaining the reward-relevant dimension (new cumin/rosemary pairing; see Methods, Table 1). In session CD Learning 2 (cumin reward-predictive), CGG mice and WT mice performed similarly well (Fig. 8*C*, *D*; HR = 1.07, 95% CI 0.54–2.11, *p* = 0.85). Also, in the following session Conflict Learning 2 (rosemary reward-predictive), CGG animal performance was not significantly different from that of WT animals (Fig. 8*C*, *D*; HR = 1.27, 95% CI 0.66–2.44, *p* = 0.46). It appears that additional training in Phase 2, performed on the same day as the preceding CD Learning 1 and Conflict Learning 1 sessions, has improved CD and conflict learning performance of CGG animals to a degree that it was now not significantly different from WT animal performance.

##### Phase 3.

The following day, Phase 3 presented animals with another all-change scenario of novel stimuli. Odor continued to be the reward-relevant dimension in an oregano/nutmeg pairing. In session CD Learning 3 (oregano reward-predictive), CGG and WT animals performed similarly in terms of ETC and TTC (Fig. 8*E*, *F*; HR = 1.58, 95% CI 0.99–2.51, *p* = 0.0504). However, in the following session Conflict Leaning 3 (nutmeg reward-predictive), CGG animals performed significantly worse than WT animals (Fig. 8*E*, *F*; HR = 0.42, 95% CI 0.19–0.93, *p* = 0.032). The results indicate persistently impaired cognitive control as CGG animals adhere to outdated and therefore inappropriate response strategies.

##### Phase 4.

Does prolonged training improve conflict learning performance of CGG animals? In Phase 4 of our ASST analysis, all stimuli were changed, and in an extradimensional shift (EDS), the digging medium became the reward-relevant dimension with a perlite/sand pairing. In session CD Learning 4 (perlite reward-predictive), CGG and WT animal performance was similar (Fig. 8*G*, *H*; HR = 0.86, 95% CI 0.67–1.10, *p* = 0.24). In the following session Conflict Leaning 4 (sand reward-predictive), we also did not observe significant differences between CGG and WT animal performance (Fig. 8*G*, *H*; HR = 0.52, 95% CI 0.24–1.15, *p* = 0.11). The results indicate that in Phase 4, conflict learning performance has improved as a result of extended training. The ASST assesses prefrontal cortical function (Brown and Bowman, 2002; Garner et al., 2006; Tait et al., 2014), and performance improvements as a result of continued training have been described for conflict learning mediated by the prefrontal cortex (Dias et al., 1997; Schoenbaum et al., 2002).

CGG animals committed significantly more errors than WT animals in Conflict Learning sessions 1 and 3 (Fig. 8). These errors were analyzed and categorized as perseverative (continuing to adhere to a previously correct but now incorrect choice) and regressive (continuing to adhere to a previously correct but now incorrect choice even after having made at least one correct choice earlier in the same Conflict Learning session; see Methods). In the latter case, the animal had regressed to a selection strategy that it had just experienced as unsuccessful (Baker et al., 2011; Amodeo et al., 2012; Iacoangeli et al., 2017).

While there was no significant difference between the number of perseverative errors committed by CGG animals and WT animals, CGG animals committed about twice as many regressive errors as did WT animals (not illustrated). The level of significance was *p* = 0.03 (*t* test) or *p* = 0.0548 (Mann–Whitney test). Since the distribution of regressive errors committed by CGG animals was skewed, the Mann–Whitney test is preferred, and we consider the CGG–WT difference in regressive errors borderline significant. Regressive cognitive impairment has also been observed in BC1 KO animals (Iacoangeli et al., 2017).

In summary, we conclude that CGG animals are impaired when confronted with a novel situational context that conflicts with previously acquired memories, and that such impairment is alleviated by extended training.

##### Spontaneous alternation.

Spontaneous alternation is an expression of innate spatial curiosity in rodents (Lalonde, 2002; Deacon and Rawlins, 2006). In a T-maze task, an animal will, after having visited one of two maze arms in a first trial, preferentially visit the respective other arm in a second trial. Performance in this task strongly relies on hippocampal spatial cognition (Deacon and Rawlins, 2006).

In previous work, BC1 KO animals did not significantly differ from WT animals in their spontaneous alternation performance (Iacoangeli et al., 2017). Here, we examined spontaneous alternation of CGG animals in a T-maze task, as described (Deacon and Rawlins, 2006; Iacoangeli et al., 2017). We failed to detect significant performance differences between CGG animals and respective WT animals (Mann–Whitney test, *p* = 0.202, 8 CGG animals and 10 WT animals; data not illustrated). We conclude that spontaneous alternation, a form of hippocampal spatial cognition, is intact in BC1 KO animals as well as in CGG animals.

In the work presented above, three behavioral approaches were applied to test a prediction resulting from our working hypothesis. The prediction was that behavioral abnormalities resulting from severely diminished presence of regulatory BC1 RNA in synapto-dendritic domains would recapitulate those resulting from global absence of the RNA in animal brains. The prediction was corroborated by the experimental results.

## Discussion

Regulatory BC RNAs, which reversibly repress translation of neuronal target mRNAs (Wang et al., 2002, 2005; Lin et al., 2008; Eom et al., 2011; 2014; Briz et al., 2017), are located to synapto-dendritic domains in WT neurons (Tiedge et al., 1991, 1993; Chicurel et al., 1993; Muslimov et al., 1997, 2006, 2011; Lin et al., 2001). In (CGG)_180_ KI animals, dendritic delivery of endogenous BC1 RNA is severely impaired, as a result of CGG-repeat competition, and the RNA remains largely restricted to perikaryal somatic areas. Absence of regulatory BC1 RNA causes early-onset phenotypic abnormalities including epileptogenic susceptibility and cognitive dysfunction (Zhong et al., 2009; Chung et al., 2017; Iacoangeli et al., 2017). We therefore reasoned that significantly reduced presence of BC1 RNA in CGG-animal synapto-dendritic domains would recapitulate such early abnormalities. Experimental scrutiny of this prediction was one of the key goals of the present work. We consider BC1 RNA mislocalization first.

### Mislocalization

**Figure 9.**
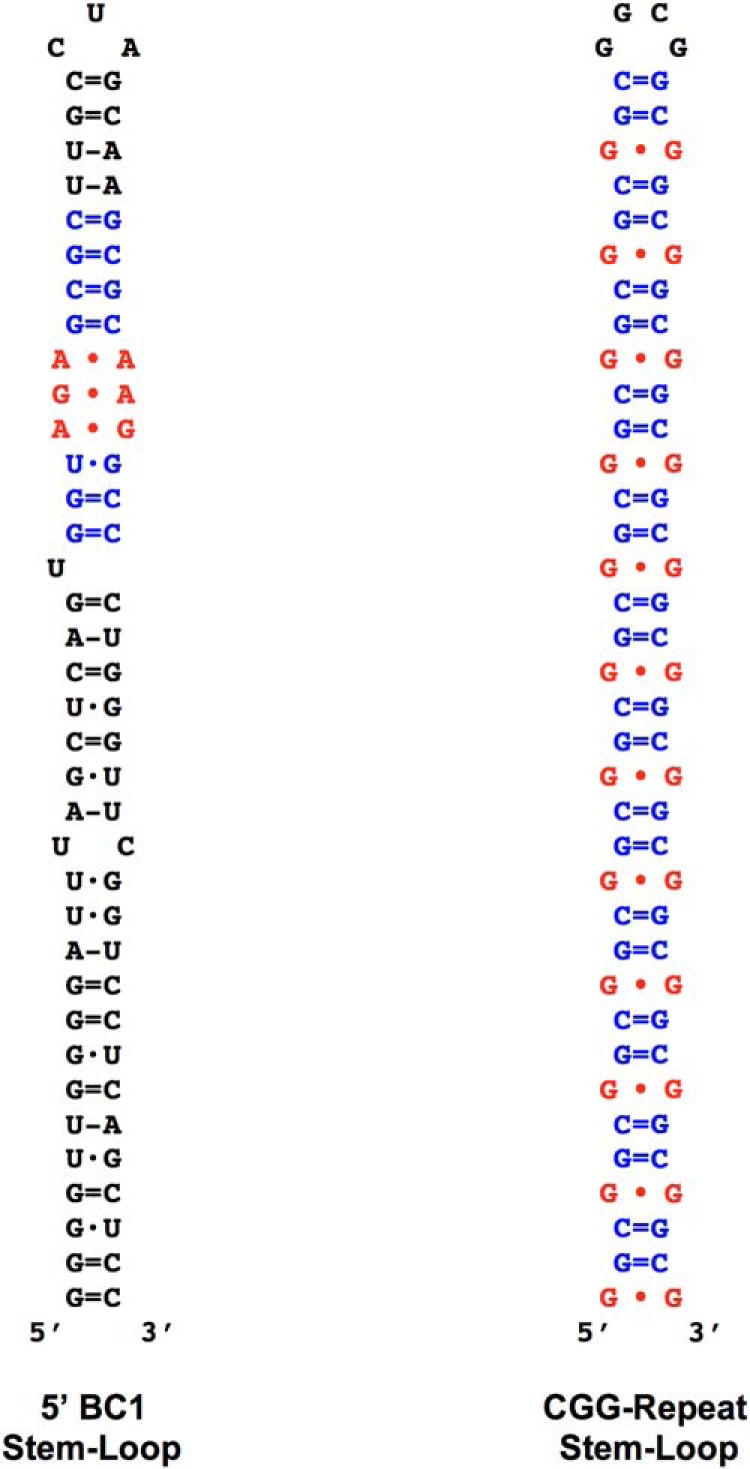
BC1 RNA DTE and CGG-repeat stem-loops: noncanonical motif structures. Noncanonical purine∙purine pairs are symbolized by ∙, standard WC pairs by = (GC) or – (AU), wobble WC pairs by ·. In the BC1 RNA DTE, the noncanonical GA core motif resides in an A-form helix that is part of the 5′ BC1 apical stem-loop domain (Muslimov et al., 2006; 2011). The GA core (red) is clamped by canonical base pairs which are mostly G=C standard WC (blue). The structure of the 5′ BC1 domain was established experimentally (Rozhdestvensky et al., 2001). In CGG-repeat stem-loops, noncanonical G∙G pairs (red) are flanked by G=C standard WC pairs (blue; Napierala et al., 2005; Zumwalt et al., 2007; Kiliszek et al., 2011). Noncanonical R∙R pairs (e.g. A∙G, G∙G) are rather strong, comparable in stability to A-U WC pairs (Mládek et al., 2009; Sobczak et al., 2010).

BC RNA DTEs reside within 5′ stem-loop structures (Muslimov et al., 1997, 2006, 2011). A 5′ DTE requirement for dendritic localization has also been confirmed using transgenic mice (Robeck et al., 2016, Table 1; but see accompanying online technical comment in Robeck et al., 2016, and Eom et al., 2018). BC RNA DTEs express spatial codes in the form of noncanonical GA motifs; such motifs feature purine∙purine interactions in which tandem G∙A/A∙G pairs engage in Hoogsteen-type hydrogen bonding (Muslimov et al., 2006, 2011; Iacoangeli and Tiedge, 2013; Eom et al., 2018; Fig. 9). The noncanonical purine∙purine base pairs are flanked (“clamped”) by standard WC G=C pairs (Fig. 9). CGG expanded repeats also form stem-loop structures in which noncanonical G∙G pairs, engaging in Hoogsteen-type interactions, are flanked by standard WC G=C pairs (Napierala et al., 2005; Kiliszek et al., 2011; Fig. 9). It is this structural equivalence, we suggest, that enables recognition by RNA transport factor hnRNP A2 of both BC RNA DTE stem-loops and expanded CGG-repeat RNA stem-loops (Muslimov et al., 2006, 2011; Sofola et al., 2007; Swanson and Orr, 2007). It is this dual recognition by hnRNP A2 that forms the basis of CGG-repeat competition with BC RNAs.

The above considerations prompt the question whether dendritic RNAs in addition to regulatory BC RNAs may similarly engage with hnRNP A2 and may thus be subject to CGG-repeat competition as well. This does not seem to be the case for dendritic MAP2 mRNA as it localizes normally in CGG animal brains (Fig. 4). Although MAP2 mRNA interacts with hnRNP A2 (Shan et al., 2003), it is not known whether a GA-motif or a similar stem-loop structure is supporting this interaction. Furthermore, other transport factors, in addition to hnRNP A2, may participate in the dendritic localization of MAP2 mRNA. For instance, trans-acting RBPs MARTA1 and MARTA2 have been identified as interacting with the DTE contained within the 3′ UTR of MAP2 transcripts (Rehbein et al., 2000, 2002). Given these considerations, we submit that regulatory BC RNAs are differentially exposed to CGG-repeat competition. Future work will reveal whether CGG-repeat competition for transport factor hnRNP A2 is uniquely affecting dendritic delivery of regulatory BC RNAs or whether, in addition, other dendritic RNAs are impacted.

As discussed above, the near-complete lack of BC1 RNA in CGG-brain synapto-dendritic layers prompted the prediction, to be considered in the following, of BC1 KO–like early-onset abnormalities including hyperexcitability and cognitive impairment.

### Physiology and cognition

**Figure 10.**
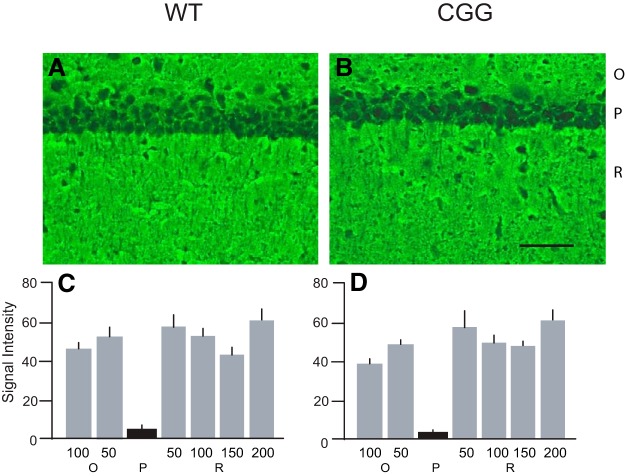
Somato-dendritic distribution of synaptophysin in WT and CGG brains. ***A***, ***B***, Synaptophysin distribution in hippocampal CA1 of WT and CGG animals. CA1 strata oriens (O), pyramidale (P), radiatum (R) are indicated. Scale bars, 100 μm. ***C***, ***D***, Quantitative analysis: one-way ANOVA, *p* = 0.92571. Comparison of signal intensities (given in relative units) in stratum P (center), in stratum O at distances of 50 and 100 μm from edge of stratum P, and in stratum R at distances of 50, 100, 150, and 200 μm from edge of stratum P, between WT and CGG animals: *p* > 0.05 for all sample points. *n* = 4 for WT and CGG.

For BC1 KO animals, epileptogenic susceptibility has been reported at an age of 18–21 d (Zhong et al., 2009) and cognitive impairment at an age of 8–12 wk (Iacoangeli et al., 2017). These findings provided motivation to test the prediction that similar early-onset phenotypic alterations would manifest in CGG animals of corresponding age groups. Previous CGG animal phenotypic analyses have typically focused on older animals, reporting FXTAS-like late-onset deficits such as intranuclear neuronal inclusions and neuromotor disturbances (Willemsen et al., 2003; Van Dam et al., 2005). In contrast, histological alterations were not detected in CGG animals younger than 20 wk (Willemsen et al., 2003). We performed additional experiments to scrutinize the histological status of synapto-dendritic regions in young (12 wk) CGG animals. Using immunohistochemistry with an antibody against the synaptic vesicle protein synaptophysin (Tiedge and Brosius, 1996; Muslimov et al., 1998), we were unable to detect any significant differences in intensity or distribution of synaptophysin labeling between CGG and WT CA1 (Fig. 10). In addition, we found that dendritic localization of MAP2 protein does not differ between CGG animal hippocampus and WT animal hippocampus (data not illustrated). The absence of histological abnormalities provides further indication of phenotypic specificity at the level of BC1 RNA mislocalization.

In hippocampal slice preparations from 18–21-d-old CGG animals, but not in matched WT animal preparations, prolonged synchronized discharges were recorded in CA3 principal neurons after synaptic activation of group I mGluRs. Blockade of group I mGluRs converted such epileptiform discharges back to short synchronized bursts. We conclude that neuronal hyperexcitability triggered by synapto-dendritic reduction of BC1 RNA manifests in the same type of prolonged epileptiform discharges as does hyperexcitability triggered by global lack of BC1 RNA (Zhong et al., 2009).

In vivo, susceptibility to audiogenic seizures was observed in 18–21-d-old CGG animals at a rate and in a manner similar to those in young BC1 KO animals (Zhong et al., 2009). In both animal models, audiogenic seizure activity is dependent on group I mGluR activation and de novo protein synthesis. BC1 RNA is counteracting stimulation of neuronal protein synthesis resulting from group I mGluR activation (Zhong et al., 2009; Iacoangeli and Tiedge, 2013; Eom et al., 2018); therefore, when BC1 RNA translational control is lacking or insufficient, group I mGluR signaling and protein synthesis has to occur for exaggerated excitability to manifest. It is also noted that BC1 RNA is expressed at high levels in the auditory system, including neuropil areas of the inferior colliculus and the auditory cortex (Lin et al., 2001).

Cognitive-behavioral deficits of young-adult CGG animals also recapitulate those of BC1 KO animals (Iacoangeli et al., 2017): excessive self-grooming and cognitive impairment, i.e., failure to disengage from memorized but situation-conflicting information. Unlike WT animals, CGG animals display persistently impaired cognitive flexibility, as they adhere to previously established outcome expectancies even after experiencing adverse actual outcomes. Such cognitive inflexibility is not the result of an underlying learning or memory disability, as CGG animals were performing well in sessions preceding and following conflict learning sessions (e.g., Conflict Learning 3). Rather, CGG animals (like BC1 KO animals) appear impaired in their flexible, context-appropriate use of stored memories.

Similar cognitive inflexibility has been observed in human ASD (Ozonoff et al., 1994; D’Cruz et al., 2013). Our data also echo clinical observations with young fragile X premutation carriers as some of these present with ASD symptoms (Goodlin-Jones et al., 2004; Farzin et al., 2006; Clifford et al., 2007; Hagerman et al., 2011, 2016).

A key goal of this paper was the test of the prediction that absence, or significantly reduced presence, of regulatory BC1 RNA in synapto-dendritic domains would engender phenotypic abnormalities identical or similar to those resulting from global absence of the RNA. This prediction was corroborated experimentally. We further established that such abnormalities, hyperexcitability and ASD-like cognitive dysfunction, manifest early-onset in young-adult CGG animals. Subsets of human premutation carriers also exhibit early-onset neurodevelopmental dysfunction that may include epilepsy, ASD, and related cognitive impairment (Jacquemont et al., 2007; Hagerman et al., 2010, 2016; Chonchaiya et al., 2012; Hagerman, 2013). Early-onset disturbances contrast with FXTAS-like late-onset manifestations, e.g., neuronal inclusions and motor dysfunction which in CGG animals are not observed before 20 wk or 1 yr of age, respectively (Willemsen et al., 2003; Van Dam et al., 2005). The question of how early- and late-onset alterations are causally and mechanistically entwined will remain a challenge for current and future premutation research (Hagerman et al., 2016).

The fragile X premutation disorder may be of multifactorial causality (Hagerman, 2013), as previous reports have advanced various molecular-cellular deficits as potentially underlying causes. Proposed mechanisms include sequestration of various RBPs, among them hnRNP A2, Purα, Sam68, DROSHA, and DGCR8 (Jin et al., 2007; Sofola et al., 2007; Sellier et al., 2010, 2013), and CGG repeat–associated translation (Todd et al., 2013; Kearse et al., 2016; Sellier et al., 2017). Phenotypic outcomes will depend on molecular specifics, e.g., functional roles of sequestered RBPs or downstream sequelae of CGG-repeat translation. Such mechanisms are not necessarily mutually exclusive, and among the open questions to be addressed will be the possibility that they may in fact be interacting.

We conclude by emphasizing that the work presented here does not, and cannot, constitute proof of the hypothesis that BC RNA mislocalization is the cause of fragile X premutation phenotypic manifestations. Inasmuch as definitive proof of any hypothesis is impossible on theoretical grounds (Popper, 1934), the current work provides in vitro and in vivo evidence that is consistent with the above hypothesis. Further tests will be needed, and future work will dissect the molecular pathway hypothesized to link CGG repeat–induced RNA mislocalization to epilepsy and cognitive impairment. Such work will also reveal whether CGG-repeat competition for transport factor hnRNP A2 is uniquely affecting dendritic delivery of regulatory BC RNAs or whether other dendritic RNAs are impacted as well.

## Synthesis

Reviewing Editor: Alfonso Represa, INSERM Decisions are customarily a result of the Reviewing Editor and the peer reviewers coming together and discussing their recommendations until a consensus is reached. When revisions are invited, a fact-based synthesis statement explaining their decision and outlining what is needed to prepare a revision will be listed below.

Dear authors,

I have been reviewing your manuscript, the comments of J Neurosci referees, your rebuttal and your corrections.

I think that a point all of us agree is that in the present status, your work identifies a hypothesis that is not fully demonstrated: that the behavioral deficit arises from impaired localization of BC1 RNA. Despite this, your data are interesting and I think they deserve publication in eNeuro. Consequently, some changes and corrections are required before acceptation. I propose you to amend the title that is somehow misleading, as the link between the mislocalization and the phenotype is not proven. I am also asking you to modify your discussion for including a paragraph stating clearly the limitation of your work in an explicit way, much in line with the comments of the referees, so that future readers will not be misled or will not over-interpret the study. I also think that the ‘Abstract’ and the ‘Significant statement’ “must be less affirmative (last sentences) for the same reasons”.

Sincerely yours
